# Limited data exist to inform our basic understanding of micronutrient requirements in pregnancy

**DOI:** 10.1126/sciadv.abj8016

**Published:** 2021-10-22

**Authors:** Emily R. Smith, Siran He, Kevin C. Klatt, Matthew D. Barberio, Ali Rahnavard, Negeena Azad, Carolyn Brandt, Bethany Harker, Emily Hogan, Padmini Kucherlapaty, Dina Moradian, Alison D. Gernand, Homa K. Ahmadzia

**Affiliations:** 1 Department of Global Health, The Milken Institute School of Public Health, The George Washington University, Washington , DC 20052 USA.; 2 Department of Exercise and Nutrition Sciences, The Milken Institute School of Public Health, The George Washington University, Washington, DC 20052 USA.; 3 USDA Children’s Nutrition Research Center, Baylor College of Medicine, Houston, TX 77030, USA.; 4 Computational Biology Institute, Departments of Biostatistics and Bioinformatics, The Milken Institute School of Public Health, The George Washington University, Washington, DC 20052, USA.; 5 Department of Nutritional Sciences, The Pennsylvania State University, University Park, PA 16801, USA.; 6 Division of Maternal-Fetal Medicine, Department of Obstetrics and Gynecology, The George Washington University School of Medicine and Health Sciences, Washington, DC 20052, USA.

## Abstract

Women and pregnant people have historically been underrepresented in research; this may extend to the basic research informing nutrient reference values, such as the United States’ and Canada’s Dietary Reference Intakes (DRIs). After screening the DRI reports for 23 micronutrients, we extracted metadata from 704 studies. Women were excluded in 23% of studies, and they accounted for a smaller proportion of the sample size (29%). Pregnant or lactating people were included in 17% of the studies. Studies that used rigorous design elements, such as controlled feeding and stable isotope studies, were the most likely to include men only. The majority of studies (>90%) did not report race and ethnicity. Although nutrient reference values are intended for use in the general population, we find that the basic science informing these values may not be generalizable. We call urgently upon funders and researchers to address fundamental gaps in knowledge with high-quality research.

## Teaser

Women and pregnant people are missing from high-quality research that informs nutrient reference values.

## INTRODUCTION

Women and pregnant people have historically been underrepresented in medical research. More than 90% of clinically approved drugs lack appropriate information on efficacy, safety, teratogenicity, and pharmacokinetics in pregnancy (*1*). While some may argue that this is a historical problem, we have witnessed the same pattern early in the coronavirus disease 2019 (COVID-19) crisis; less than 2% of all COVID-19 registered trials included pregnant people (*2*). Even in COVID-19 treatment trials using medications or micronutrients with known safety profiles, most of the protocols listed pregnancy as an exclusion criterion (*3*). The justifications for excluding pregnant people from research center on protection of the fetus and perceived logistical complications of recruitment in pregnancy (*4*, *5*). However, we join many bioethicists to argue that pregnant people should be protected through research rather than from research (*5*).

Exclusion of women from high-quality medical research or failure to rigorously consider sex as a biologic variable is problematic because there is likely to be sexual dimorphism in the physiology, metabolism, and related efficacy and toxicity of supplements and drugs (*6*). A 2001 U.S. Food and Drug Administration (FDA) report found that 8 of the 10 drugs recalled by the FDA from 1997 to 2000 posed greater health risks for females as compared with males (*7*). Given the physiologic adaptations that occur over the course of pregnancy, there is further biological imperative to specifically study any drugs, xenobiotics, or supplements that ultimately affect the processes of absorption, distribution, metabolism, and excretion (*8*). The study of nutrition during pregnancy is particularly critical given the emerging literature that demonstrates life-long impacts of maternal diet and nutritional status on fetal health and the offspring’s future health (*9*, *10*). Thus, there is both an ethical and biological imperative to include pregnant people in nutrition research.

Nutrient reference values (NRVs) play important roles in the public and private sectors. Four basic indicators typically comprise NRVs (Supplementary Text). Governments and public health agencies use NRVs broadly to develop food-based dietary guidelines and to assess and monitor the adequacy of population nutrient intakes. The private sector uses NRVs to develop food products and to determine dietary supplement composition. The establishment of and continued updates to NRVs are determined by various national and international agencies. The Institute of Medicine of the National Academies (now the National Academies of Science, Engineering, and Medicine) established NRVs for the United States and Canada. These are called the Dietary Reference Intakes (DRIs) (*11*), which include the following components: the estimated average requirement (EAR), the recommended dietary allowance (RDA, derived from the EAR), the adequate intake (AI), and the tolerable upper intake level (UL, hereafter referred to as “upper level”). These values are set for healthy populations, and despite their intended use in North America, they are widely adopted globally for policy setting, public health programming, and commercial references. However, the evidence underlying these NRVs may not be representative of racial or ethnic minorities and people living in low- and middle-income countries (LMICs).

The objective of this study was to critically appraise the evidence base informing the NRVs of 23 selected micronutrients. We investigated the population included and methods used in the studies informing the development of these values, specifically the selection of indicators and the reference values related to the population average requirements (EAR/RDA) and upper levels, which are the core reference values (*12*). We assessed the following: (i) the extent to which women, pregnant or lactating people, racial or ethnic minorities, and people living in LMICs were included in the studies cited in relevant sections of the DRI reports; (ii) whether people classified as having poor nutritional or health status were included, given global variability in health status; and (iii) the extent to which studies used rigorous, molecular, and modern methods.

## RESULTS

### Characteristics of included studies

**Fig. 1.**
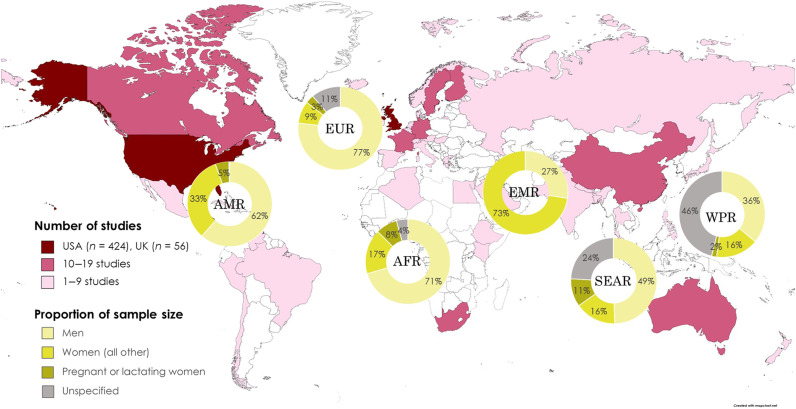
Number of research studies informing the micronutrient reference values by country and proportion of research subjects by sex for each World Health Organization region. Created with www.mapchart.net. AFR, African Region; AMR, Region for the Americans; EMR, Eastern Mediterranean Region; EUR, European Region; SEAR, South-East Asia Region; WPR, Western Pacific Region.

We reviewed 2320 studies across five DRI reports, and we ultimately included 704 primary research studies that contributed to setting the NRVs: 238 in the “indicator” section (which included scientific background on selecting key indicators to assess the status of each micronutrient), 347 in the “life stages” section (which contain information on the average requirements for a healthy population in each life stage group including pregnancy and lactation), and 119 in the “upper level” section (which provided scientific basis of setting the highest average daily nutrient intake level that is likely to pose no health risk to almost all individuals in the general population) (fig. S1). A total of 424 (60.2%) studies were conducted in the United States and 56 (8.0%) in the United Kingdom (Fig. 1). Only 8.2% of the included studies were conducted in LMICs (table S1). More than 60% of the included studies were open access, a feature that has recently become more common (fig. S2).

### Study populations in human studies

A total of 671 (95.3%) of the included studies were conducted among human participants (table S1), and of those 77.5% included women (Table 1). Male-only studies accounted for 16.5% of the studies. Among studies where sex was reported, women accounted for about 28.9% of all study participants (Table 1). The number of studies and proportion of the study population reported as women varied across DRI report sections. Women were included the most in studies cited in the life stages section (56.3% of the study population) and were least likely to be included in the studies used to inform the upper level section (23.1% of the study population).

**Table 1.** Number of human studies and sample size by participant sex overall and for each DRI report section.

**Characteristics**	**Section in the DRI reports**			
	**Indicator**	**Life stages**	**Upper level**	**Total**
***Number of studies***	***N = 233***	***N = 336***	***N = 102***	***N = 671***
Included women	157 (67.4%)	285 (84.8%)	78 (76.4%)	520 (77.5%)
Included pregnant or lactating women*	12 (5.2%)	98 (29.2%)	7 (6.9%)	117 (17.4%)
Men only	58 (24.9%)	38 (11.3%)	15 (14.7%)	111 (16.5%)
Sex unspecified	18 (7.7%)	13 (3.9%)	9 (8.8%)	40 (6.0%)

***Sample size among 640 studies reporting*** ***sample size by sex* ^†^**	***n = 674,522***	***n = 53,646***	***n = 185,997***	***n = 914,137***
Sample size of all women, *n* (%)	190,818 (28.3%)	30,220 (56.3%)	42,884 (23.1%)	263,922 (28.9%)
Pregnant or lactating women, *n* (%)^‡^	482 (0.1%)	18,144 (33.8%)	23,521 (12.6%)	42,147 (4.6%)
Sample size of all men, *n* (%)	483,704 (71.7%)	23,398 (43.6%)	143,113 (76.9%)	650,215 (71.1%)
Sample size of men in studies that only included men, *n* (%)^§^	404,362 (59.9%)	1,015 (1.9%)	284 (0.2%)	405,629 (44.4%)

*Studies that included pregnant or lactating women are nested within studies that included women; we combined pregnant and lactating women into the same category.

†Sample size with unspecified sex: *n* = 46,086, *n* = 782, and *n* = 4393 in the indicator, life stages, and upper level sections, respectively (total = 51,261).

‡Not all studies reported sample size for women or pregnant women, even when they were included.

§Subset of the “men” category.

**Fig. 2.**
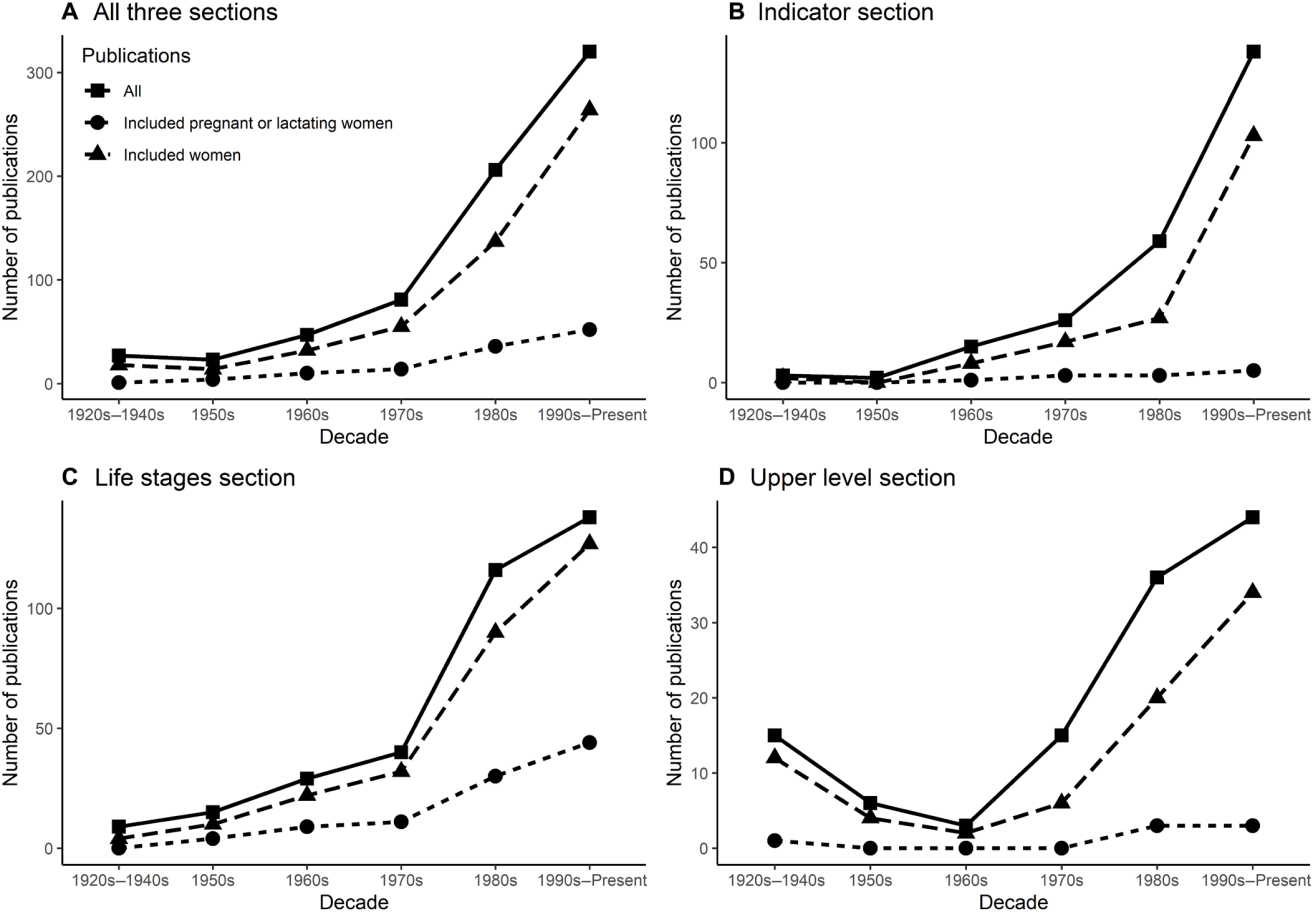
Number of studies that included women and pregnant or lactating women, by decade in the DRI reports.

Less than 20% of all studies included pregnant or lactating women. In terms of sample size, 42,147 pregnant or lactating women (4.6% of total sample size) were included in the studies, with only 482 (<0.1% of the population) in the indicator section (Table 1). We also observed that, for 16 of 23 micronutrients, the reports did not consider any study including pregnant or lactating people in selecting key indicators, and 18 micronutrients had no pregnancy or lactating data in setting the UL (Table 2). The percentages of studies that included women and pregnant or lactating women varied by micronutrient (fig. S3). Despite the sharp increase in relevant publications beginning in the mid-20th century, there was no corresponding increase in the proportion of studies that included pregnant or lactating women (Fig. 2 and fig. S4).

**Table 2.** Number of human studies including women and pregnant or lactating women, by DRI report section and by micronutrient. The order of the micronutrients: alphabetical for vitamins first, followed by minerals. Heatmap color gradient from red, pink, to white corresponds from lowest to highest proportions of studies. When the denominators were zero in a given section for a micronutrient, we presented the percentages as 0% instead of “incalculable.”

**Micronutrient**	**Total** **number** **of studies**	**Indicator section**			**Life stages section**			**Upper level section**		
		**Number** **of studies**	**Included** **women (%)**	**Included** **pregnant** **or lactating** **women (%)**	**Number** **of studies**	**Included** **women (%)**	**Included** **pregnant** **or lactating** **women (%)**	**Number** **of studies**	**Included** **women (%)**	**Included** **pregnant** **or lactating** **women (%)**
Vitamin A	17	3	67%	0%	9	89%	33%	5	100%	40%
Vitamin B1 (thiamin)	17	7	57%	0%	10	60%	10%	0	0%	0%
Vitamin B2 (riboflavin)	33	10	70%	10%	23	87%	30%	0	0%	0%
Vitamin B3 (niacin)	15	2	0%	0%	5	80%	0%	8	50%	0%
Vitamin B5 (pantothenic acid)	10	3	33%	0%	7	100%	71%	0	0%	0%
Vitamin B6 (pyridoxine)	38	17	65%	18%	17	94%	24%	4	75%	0%
Vitamin B7 (biotin)	11	10	70%	0%	1	100%	0%	0	0%	0%
Vitamin B9 (folate)	99	33	64%	12%	46	80%	33%	20	80%	5%
Vitamin B12 (cobalamin)	56	9	100%	0%	47	74%	15%	0	0%	0%
Choline	13	5	80%	0%	4	75%	0%	4	75%	0%
Vitamin C	10	1	0%	0%	8	100%	13%	1	100%	0%
Vitamin D and calcium	70	33	100%	0%	28	100%	32%	9	67%	0%
Vitamin E	16	1	0%	0%	10	80%	20%	5	80%	0%
Vitamin K	10	0	0%	0%	10	100%	20%	0	0%	0%
Carotenoids	33	33	48%	0%	0	0%	0%	0	0%	0%
Copper	15	4	75%	0%	6	50%	17%	5	80%	0%
Iodine	25	16	69%	6%	5	80%	40%	4	75%	0%
Iron	35	12	83%	17%	15	87%	13%	8	88%	25%
Magnesium	44	8	38%	0%	26	100%	58%	10	90%	10%
Phosphorus	15	1	0%	0%	9	100%	89%	5	60%	0%
Selenium	20	5	100%	20%	9	78%	22%	6	83%	0%
Zinc	69	20	50%	0%	41	78%	29%	8	63%	13%
Total (all micronutrients)	671	233	67%	5%	336	85%	29%	102	76%	7%

Across the six World Health Organization regions, women represented one-third of the study population (Fig. 1). The lowest proportion of female participants was for studies conducted in the European region (including the United Kingdom), whereas the highest proportion was in the Eastern Mediterranean Region. In terms of pregnant or lactating women, the sample size ranged from 0.0 to 12.0% across all regions. In the African Region and the European Region, men accounted for more than 70.0% of the study populations.

Less than one-third of the human studies reported the race or ethnicity of study participants (Table 3). Of the studies conducted in the United States, 32.0% reported race or ethnicity. Among the 555,329 participants of U.S.-based studies, 91.7% had unknown or unspecified race and ethnicity. Among the non-U.S. studies, 19.9% reported some information on race or ethnicity but often in ways that could not be grouped across studies. For example, one study reported “all South Indian”. Over half (57.2%) of the included human studies were conducted in healthy populations (Table 3).

**Table 3.** Reporting of race or ethnicity in the included human studies.

**Characteristics**	**Section in the DRI reports**			
	**Indicator** **(studies = 233)**	**Life stages** **(studies = 336)**	**Upper level** **(studies = 102)**	**Total** **(studies = 671)**
**Number of studies**				
Healthy population only (% among all studies)	122 (52.4%)	221 (65.8%)	41 (40.2%)	384 (57.2%)
Number of studies reporting race/ethnicity (% among all U.S. studies)*	37 (27.8%)	68 (33.0%)	25 (37.3%)	130 (32.0%)
Number of studies reporting race/ethnicity (% among all non-U.S. studies^†^)	15 (15.0%)	33 (25.2%)	5 (13.9%)	53 (19.9%)
				
**Number of participants among U.S. studies^‡^**				
White/Caucasian sample size, *n* (%)	4830 (1.2%)	3187 (9.9%)	17,471 (14.1%)	25,488 (4.6%)
Black/African-American sample size, *n* (%)	4150 (1.0%)	2408 (7.5%)	2751 (2.2%)	9309 (1.7%)
Asian-American sample size, *n* (%)	8216 (2.1%)	48 (0.1%)	0	8264 (1.5%)
Hispanic/Latino sample size, *n* (%)	1696 (0.4%)	179 (0.6%)	872 (0.7%)	2747 (0.5%)
Indigenous American sample size, *n* (%)	0	101 (0.3%)	0	101 (0.02%)
Race unspecified sample size, *n* (%)	380,859 (95.3%)	26,133 (81.5%)	102,428 (82.9%)	509,420 (91.7%)

*Studies conducted in the United States alone or United States in collaboration with the following: Bangladesh; Belgium and Germany; China; Cuba; Nepal; and Vietnam, Iran, and Ethiopia. Number of studies: 133, 206, 67 for indicator, life stages, and upper level, respectively.

†Studies not conducted in the United States alone, which have some overlap with the “United States” category (four studies). Number of studies: 101, 135, 47 for the three sections, respectively.

‡Sample sizes of study participants in the United States: 399,751, 32,056, and 123,522 for the indicator, life stages, and upper level sections, respectively (total, 555,329). Not all studies reported sample size for each race or ethnicity, even when they were reported.

### Subjects in nonhuman studies

A total of 39 studies either were conducted only in animals or reported both human and animal outcomes (table S2). About one in three (35.9%) animal studies included female animals, with a sample size of 588 (37.6% of all included animals). No pregnant animals were included in either the indicator or the upper level section. More than half of the studies reported some shared conditions of the animals, such as cobalamin replete bats.

### Study methods in human and nonhuman studies

**Fig. 3.**
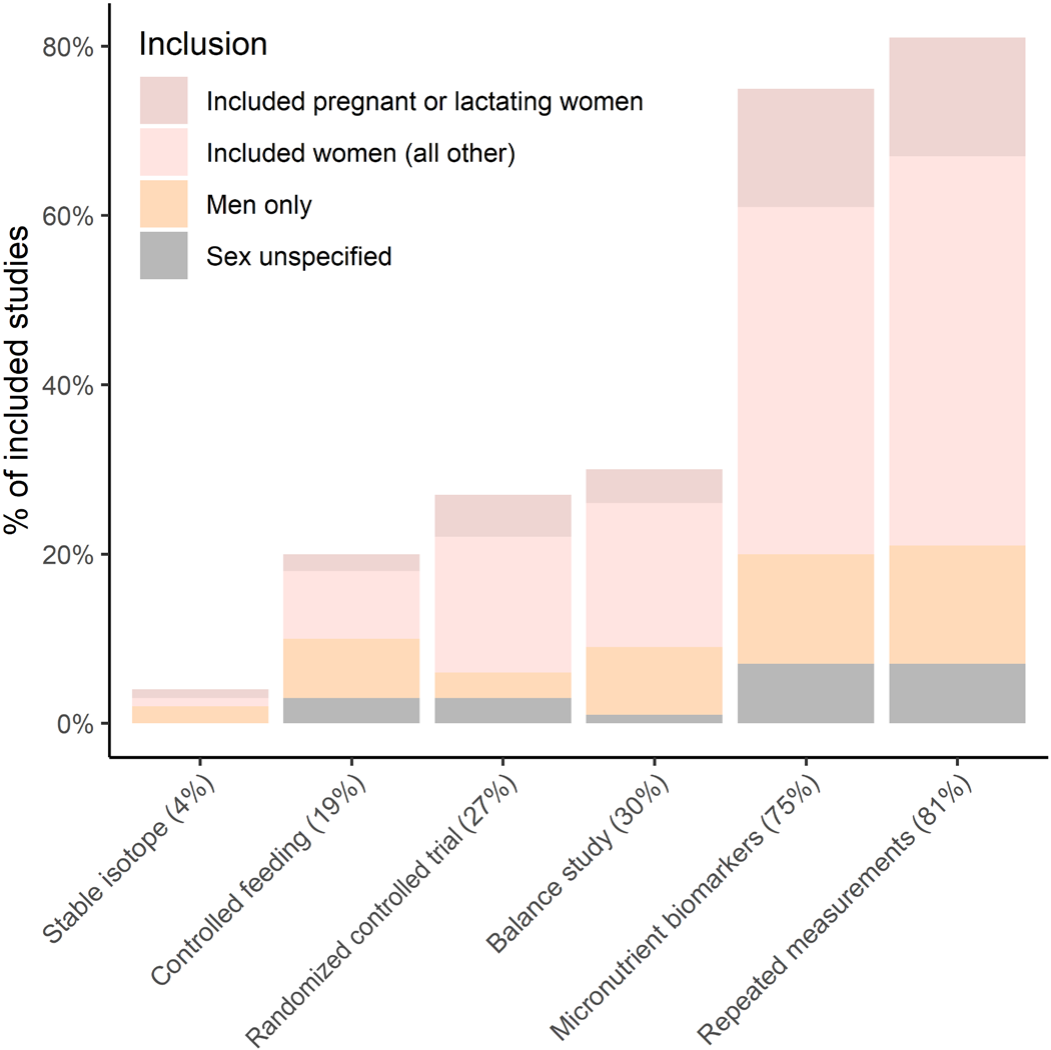
Percentage of studies using specific rigorous methods, stratified by sex and life stage. The percentages on the *x*-axis labels refer to the proportion of human studies that included each method.

Just over one-fourth (26.8%) of the included studies were randomized controlled trials (RCTs) (Fig. 3 and table S3). One-fifth (19.3%) were controlled feeding studies, 30.0% were balance studies, and 80.5% included repeated measurements. One hundred and twenty (17.0%) studies included none of these elements of rigorous design. With regard to molecular methods, 4.4% of the studies used stable isotopes for the respective micronutrient. Seventy-five percent of the included studies measured biomarkers for micronutrients; the highest proportion was in the indicator section (83.2%). We assessed whether genomic, metabolomic, or proteomic methods were used and found only one study using genomics methods (*13*).

Among the studies with rigorous design, 78.3% of RCTs included women, 17.5% included pregnant or lactating women, and 11.1% included men only (Fig. 3). Among controlled feeding studies, 36.0% involved only men, whereas men-only studies were observed in 35.5% of studies with stable isotopes, 26.5% of balance studies, 17.4% of studies that had micronutrient biomarkers, and 17.3% of studies with repeated measurements.

### Setting the DRIs for pregnancy

The NRVs for pregnancy were determined on the basis of different evidence for each micronutrient, although a factorial approach was most commonly used (table S4). For example, the estimated average requirement for thiamin in pregnancy was increased by one-third on top of the recommended level in adult females, which accounts for approximated maternal and fetal needs during pregnancy, and was not based on experimental data. In contrast, the average requirement of calcium for pregnant women was determined to be the same as that of nonpregnant females, which was based on RCTs of calcium supplementation during pregnancy. Adverse pregnancy health outcomes such as neural tube defects or fetal growth restriction were not selected as indicators for setting the pregnancy reference values (table S4).

### Sources of research funding

Among the included studies, 498 (70.7%) reported funding (table S5). Of the 326 studies that received funding solely from the government, 240 (73.6%) included women, and 54 (16.6%) included pregnant or lactating women. Thirty-one studies received funding only from private industry, and 28 (90.3%) included women, whereas 6 (19.4%) included pregnant or lactating women. Of the 44 studies that received support from foundations or philanthropic organizations, 31 (70.5%) and 3 (6.8%) reported the inclusion of women and pregnant or lactating people, respectively.

## DISCUSSION

We found that women have been historically underrepresented in nutrition research that informs the DRIs. Inclusion of women has occurred to an even lesser extent in studies using state-of-the-art nutrition research methods including controlled feeding and balance studies, as well as studies using stable isotopes. We also observed that pregnant and lactating people have been missing from clinical nutrition research that informs the NRVs for these crucial life stages. In animal studies, pregnant and nonpregnant females were also underrepresented. On the basis of our nutrient-specific summary of how the dietary reference intake values were established, most of the reference values for pregnancy were determined by modifying the values for nonpregnant women using a modeling method, and sometimes the values for women were derived from men. We therefore reported the number of studies including pregnant people as a proportion of all studies in the adult life stages sections (which formed the evidence basis for setting the average requirements or EARs/AIs), despite that one would not expect pregnant or lactating people to be included in all research studies. We also found that pregnancy health outcomes were not selected as indicators for any micronutrient and thus were not used to set pregnancy reference values. For example, preventing neural tube defects is not used in determining folate requirements, and preventing preeclampsia is not used in determining calcium requirements; the reports cite insufficient, high-quality data.

When we investigated the studies by each micronutrient, we observed that for most micronutrients, there was not a single study that included pregnant or lactating people to inform the selection of indicators or to establish the upper intake level. Pregnant or lactating people account for less than 1% of the total population in the studies that informed indicator selection. This is problematic because optimal biomarkers of micronutrient status may differ by reproductive state. For example, the only controlled feeding study examining vitamin B12 in pregnancy showed alterations in biomarkers, relative to nonpregnant women consuming equivalent diets, and suggested that serum holotranscobalamin or the holotranscobalamin–to–vitamin B12 ratio may be a better biomarker of vitamin B12 status in pregnancy than serum B12 (*14*). A review of European NRVs raised similar concerns about the lack of primary data—in addition to inconsistent methods to extrapolate data—for infants, children, adolescents, and pregnant or lactating women (*15*).

We were unable to conclude how racial and ethnic minority populations were represented in this field of research because more than 90% of studies did not report on the race or ethnicity of the population. This is a notably lower level of race and ethnicity reporting than in a review of clinical trials published from 1989 to 2000, which found that 41% of cardiovascular disease, diabetes, HIV/AIDS, and cancer trials failed to report on the race of participants (*16*). At a minimum, reporting the race and ethnicity of participants is essential to understand the generalizability of research to the entire population.

We postulate that underrepresented people or conditions are also the least likely to receive benefits from methodological advancements in the early stages of relevant application. In this study, we found that controlled feeding studies, balance studies, and research using stable isotopes were the least common methods used. Despite being essential methods for identifying pregnancy-specific alterations in micronutrient requirements, these methods were also the least likely to include pregnant people. We also observed that factorial methods were commonly used to set NRVs for the pregnancy life stage. For example, the AI value for choline among nonpregnant women was derived (based on body weight adjustments) from a study that focused on preventing liver dysfunction in men; the AI for pregnant women was subsequently extrapolated using a factorial approach based on the fetal and placental accumulation of choline. On the basis of this approach, a small increase (25 to 50 mg) to the nonpregnant NRV was added to set the pregnancy reference value (*17*). However, decades of animal research and a small body of human literature indicate that substantially higher intakes of choline are required for optimal fetal neurodevelopment and infant cognition (*18*, *19*). We contend that rigorous study designs and modern molecular methods can and should be used in pregnant and lactating people. While radioactive isotopes cannot safely be used in pregnancy, other nuclear methods including stable isotopes and magnetic resonance spectroscopy are suitable for studying micronutrients in pregnant and lactating women (*20*, *21*).

Less than 10% of the studies included in the DRI reports were conducted in LMICs. This is perhaps not unexpected because these are reference values intended for use in North America. However, we suspect that this reflects the state of existing evidence from LMICs, because the DRIs are one of the major, systematic efforts including high-quality data from any location. Careful consideration is needed when applying existing reference values, such as the RDA values to LMIC contexts, given the likelihood of lower nutrient intakes and potentially unique dietary and environmental exposures that may interact with nutritional status. In certain instances, reference values may be inadequate for some populations because of underestimating the need and overestimating the risk from higher-dose supplementation, such as in the case of water-soluble vitamins. In other cases, the RDA values for the United States and Canada for some micronutrients may be inappropriate to use without modifications in other contexts; such as that of iron, where bioavailability varies widely on the basis of diet and there are concerns surrounding high-dose iron supplementation in malaria endemic regions (*22*). Over the past decades, there has been evolving consensus about how best to harmonize the process of setting NRVs to allow for a globally consistent process (*12*, *23*). Part of the call to harmonize this process, and one of the benefits of doing so, is to include data from all countries and to promote global access to related data and analytical tools (*24*). However, global reference values require underlying data that are representative of the global population rather than only those living in the global north.

This study has several strengths and is subject to limitations. First, we attempt to systematically characterize the extent to which women, pregnant people, and racial and ethnic minorities have been included in the research that informs the NRVs. We used quality assurance strategies at each step of the search, screening, and data collection process. However, there are some limitations. Determining whether a study “contributed to setting the nutrient reference values” was clear in some cases but subjective in others. Further, a single researcher abstracted data for each study. However, 2% of studies were reviewed by a second reviewer, and 100% of key methods variables were confirmed by a third reviewer. Within the subset of studies that underwent double data extraction, the accuracy was high: above 90% across reviewers.

Our findings strongly support the call to action to include women and pregnant people in nutrition research. We maintain that women and pregnant people must be included in research, particularly in high-quality research with rigorous design and modern methods. Such high-quality research will require a commitment from governmental and nongovernmental agencies to fund interventions in free-living populations, controlled feeding studies, and cohort studies that include women of reproductive age and pregnant people. We echo the recommendation in the *Harmonization of Approaches to Nutrient Reference Values: Applications to Young Children and Women of Reproductive Age* that, “Researchers and funding organizations should advance the knowledge of nutrient requirement research by supporting research that uses modern technology, techniques, or methods for assessing requirements” (*12*). We further argue that sex must be considered as a biological variable in study design, data analysis, and interpretation for preclinical and clinical research, as well as in the process of establishing reference values.

The objective of meta-research in general, and of our study, is to systematically evaluate how science is produced and used (*25*). Meta-research can also help us prioritize the next steps in improving the way research is conducted. One such step might be that future DRIs committees include the metrics of diversity and inclusion calculated within this study as part of their updated reports. An additional step would be to ensure that data from male-only studies are no longer used to set reference values for all people. Any data informing reference values should be examined for sex-specific effects, although this implies that primary data will need to be reanalyzed. Future reports should consider that nutrient indicators may be specific to pregnant people as well. While it seems that many funders and researchers continue to erroneously believe themselves to be protecting women and pregnant people from research, we join with many others to urge that all people are truly protected through participation in high-quality research.

## MATERIALS AND METHODS

The detailed protocol and methods for this study were previously published (*26*). Although there are a number of NRVs set globally, we chose to focus on the DRIs for three reasons: There is an established and systematic methodology for the process, they include an EAR and UL value, and they have been updated more recently than other NRVs (*27*).

The process of establishing the DRIs aims to characterize the causal, dose-response relationship between nutrient intakes and a specific nutrient for each of the 22 life-stage groups (covering males and females across age groupings and pregnancy and lactation) (*28*). Specific indicators are selected for each nutrient; indicators generally include functional health outcomes and biomarkers of nutrient intake. When valid indicators are not available, NRVs may be derived from nutrient balance studies (assessing intake and output) and factorial estimations (i.e., intakes needed to support losses and tissue accretion) or by estimating habitual dietary intakes from a healthy population that has no apparent negative health consequences. Following the selection of an indicator, data are assessed to estimate either an EAR and RDA or an AI. The UL is established following a process established by the Institute of Medicine using a toxicological risk assessment model developed specifically for nutrients (*29*).

We reviewed the five DRI reports related to the following 23 micronutrients: vitamin A, vitamin B1 (thiamin), vitamin B2 (riboflavin), vitamin B3 (niacin), vitamin B5 (pantothenic acid), vitamin B6 (pyridoxine), vitamin B7 (biotin), vitamin B9 (folate), vitamin B12, choline, vitamin C, vitamin D, vitamin E, vitamin K, calcium, carotenoids, copper, iodine, iron, magnesium, phosphorus, selenium, and zinc (*30*–*34*). Given our focus on micronutrients, we did not review three DRI reports focused on electrolytes and macronutrients (*35*–*37*).

### Search

First, we searched for all studies that potentially contributed to the DRIs. For each micronutrient, we extracted the references of each study cited in three sections of the micronutrient-specific chapter the DRI report: “Selection of Indicators for Estimating the Requirement for [nutrient]”, “Findings by Life Stage and Gender Group”, and “Tolerable Upper Intake Levels (UL)”. The first two sections were related to setting the average requirement (EAR or AI), and the third section represents studies that contributed to setting the UL values. The indicator section provides a list of nutrient-specific indicators that were considered by the committee to estimate dietary requirements and establish NRVs, whereas the findings by life stages section provide direct evidence that supports average requirement determination. In the Findings by Life Stage and Gender Group, we extracted references for the subsections: “Adults,” “Pregnancy,” and “Lactation.” The full references were recorded by trained research assistants (C.B. and P.K.). Slight variations in the formatting of different DRI reports led to additional sections extracted for vitamin B2, vitamin B6, calcium, and vitamin D, as outlined in the previously published study protocol (*26*).

### Study selection

Next, a panel of five experts (E.R.S., S.H., K.C.K., M.D.B., and A.D.G.) with a background in nutritional science screened the titles of each study, in the context of the DRI report, to determine whether the study was ultimately used to set the DRI value based on a set of inclusion and exclusion criteria. A second reviewer (S.H.) conducted an additional round of screening and documented reasons for inclusion, exclusion, or uncertainty. Any discrepancies were resolved in discussion with a third reviewer (E.R.S.). The inclusion criteria for screening were the following: the study (i) was used to inform the determination of current DRI values (AR or UL) and (ii) presents primary data (e.g., RCT or a cohort study). Exclusion criteria were specific to each section. For the indicator section, if the DRI report directly mentioned that the indicator was not used to determine the DRIs, then studies related to that indicator were excluded. If a UL was not established for a particular nutrient, then all references in the UL section were excluded. We also excluded references in the life stage or UL sections if they were directly described in the DRI report as not being used in setting the final DRI values. For example, several references in the pregnancy section for thiamin were noted as “Data from the studies cited above are equivocal about the effects of pregnancy on thiamin requirements, and thus not useful in refining this estimate.”

### Data collection

A team of four trained research assistants retrieved and screened the full-text files of the included references (N.A., B.H., E.H., and D.M.). Studies were excluded at this stage if they were not primary research data, they were in vitro studies, the full-text file could not be found, the full-text file was illegible, the studies were in a non-English language, or the studies were duplicates. We extracted data from each study related to four domains: (i) administrative information (e.g., author names, funding sources, and location of study), (ii) study methods (e.g., rigorous methods or usage of stable isotopes), (iii) human population characteristics (e.g., number of women, number of pregnant people, and race and ethnicity of participants), and (iv) nonhuman subjects (e.g., species and breed of the animals and inclusion of female animals). The final compiled dataset can be accessed at Open Science Framework (*38*). We also summarized how the DRI values of each micronutrient were established for pregnancy based on the original text in the DRI reports. We performed quality control for each step of the review. Final quality control indicated that >95% of the postscreening articles were obtained, and the remaining could not be retrieved even with interlibrary loan requests. Data were 100% complete for retrieved full-text articles. In addition, data accuracy ranged from 91 to 94% across the four research assistants, based on a second round of independent data collection for a random subset of the studies. As discrepancies were most common for three variables related to study methods (stable isotope, controlled feeding, and balance study), a second reviewer reexamined these data.

### Data analysis and evidence synthesis

We summarized the search and study selection process overall and by section using a flowchart (fig. S1). For administrative information, we focused on the type of the publications (human or nonhuman, open access or not), the year of publication, and the context of the publication (country and context). We categorized the regions of the countries according to World Health Organization regions: African Region, Region of the Americas, South-East Asian Region, European Region, Eastern Mediterranean Region, and Western Pacific Region. Regarding study methods, we evaluated the scientific rigor in terms of RCT, controlled feeding, balance study, or repeated measurements on the same subjects (true longitudinal data). We also evaluated whether studies used molecular methods (usage of stable isotope and micronutrient biomarkers) or any modern methods such as metabolomics, proteomics, or metagenomics. We categorized study populations as men-only, women included, pregnant- and lactating-women included (we combined pregnant and lactating women because of the small sample size of the latter), or sex unspecified. We also noted whether women of reproductive age (14 to 44 years) were included and whether women-specific health outcomes were assessed (e.g., maternal anemia and preeclampsia). In addition, we summarized other characteristics of the study populations, such as race and ethnicity (separately for the U.S. and non-U.S. countries because of contextual factors) and baseline health status. We performed quantitative analysis for animal studies separately. For most of the characteristics, we focused on the means and proportions of studies. For women, pregnant or lactating women, and race and ethnicity, we also calculated the total sample size associated with these characteristics. All quantitative analyses were performed in R version 4.0.3 (R Core Team, Vienna, Austria) (*39*).

### Language

We used “women” to refer to the biologically female sex and “pregnant people” to refer to anyone who is pregnant, regardless of their gender identification. Nevertheless, in analyzing the extracted data and presenting the study results, we followed the language used in the original studies. For instance, we used “pregnant or lactating women” throughout Results but used the term pregnant people when we discussed the findings.

### Ethical approval

This meta-review study did not involve direct contact with either human or animal subjects nor did it include in vitro samples. Ethical approval was not required and thus not obtained.

## References

[1] J. Scaffidi, B. W. Mol, J. A. Keelan, The pregnant women as a drug orphan: A global survey of registered clinical trials of pharmacological interventions in pregnancy. BJOG 124, 132–140 (2017). 27297096 10.1111/1471-0528.14151

[2] D. D. Smith, J. L. Pippen, A. A. Adesomo, K. M. Rood, M. B. Landon, M. M. Costantine, Exclusion of pregnant women from clinical trials during the coronavirus disease 2019 pandemic: A review of international registries. Am. J. Perinatol. 37, 792–799 (2020). 32428965 10.1055/s-0040-1712103 PMC7356075

[3] M. M. Taylor, L. Kobeissi, C. Kim, A. Amin, A. E. Thorson, N. B. Bellare, V. Brizuela, M. Bonet, E. Kara, S. S. Thwin, H. Kuganantham, M. Ali, O. T. Oladapo, N. Broutet, Inclusion of pregnant women in COVID-19 treatment trials: A review and global call to action. Lancet Glob. Health 9, e366–e371 (2021). 33340453 10.1016/S2214-109X(20)30484-8 PMC7832459

[4] A. D. Lyerly, M. O. Little, R. Faden, The second wave: Toward responsible inclusion of pregnant women in research. Int. J. Fem. Approaches Bioeth. 1, 5–22 (2008). 19774226 10.1353/ijf.0.0047 PMC2747530

[5] Committee on Ethics, ACOG Committee Opinion No. 646: Ethical considerations for including women as research participants. Obstet. Gynecol. 126, e100–e107 (2015). 26488521 10.1097/AOG.0000000000001150

[6] A. J. McGregor, The effects of sex and gender on pharmacologic toxicity: Implications for clinical therapy. Clin. Ther. 39, 8–9 (2017). 28034520 10.1016/j.clinthera.2016.12.007

[7] Drug Safety, Most drugs withdrawn in recent years had greater health risk for women, *United States General Accounting Office* (2001).

[8] J. M. Kazma, J. van den Anker, K. Allegaert, A. Dallmann, H. K. Ahmadzia, Anatomical and physiological alterations of pregnancy. J. Pharmacokinet. Pharmacodyn. 47, 271–285 (2020). 32026239 10.1007/s10928-020-09677-1 PMC7416543

[9] J. C. King, Physiology of pregnancy and nutrient metabolism. Am. J. Clin. Nutr. 71, 1218S–1225S (2000). 10799394 10.1093/ajcn/71.5.1218s

[10] S. He, A. D. Stein, Early-life nutrition interventions and associated long-term cardiometabolic outcomes: A systematic review and meta-analysis of randomized controlled trials. Adv. Nutr. 2, 461–489 (2020). 10.1093/advances/nmaa107 PMC8009753 33786595

[11] Institute of Medicine, *Dietary Reference Intakes: The Essential Guide to Nutrient Requirements* (National Academies Press, 2006).

[12] National Academies of Sciences, Engineering, and Medicine; Health and Medicine Division; Food and Nutrition Board; Committee on the Application of Global Harmonization of Methodological Approaches to Nutrient Intake Recommendations for Young Children and Women of Reproductive Age, *Harmonization of Approaches to Nutrient Reference Values: Applications to Young Children and Women of Reproductive Age* [National Academies Press (US), 2018]. 30222283

[13] R. A. Jacob, D. M. Gretz, P. C. Taylor, S. J. James, I. P. Pogribny, B. J. Miller, S. M. Henning, M. E. Swendseid, Moderate folate depletion increases plasma homocysteine and decreases lymphocyte DNA methylation in postmenopausal women. J. Nutr. 128, 1204–1212 (1998). 9649607 10.1093/jn/128.7.1204

[14] S. Bae, A. A. West, J. Yan, X. Jiang, C. A. Perry, O. Malysheva, S. P. Stabler, R. H. Allen, M. A. Caudill, Vitamin B-12 status differs among pregnant, lactating, and control women with equivalent nutrient intakes. J. Nutr. 145, 1507–1514 (2015). 25995278 10.3945/jn.115.210757

[15] S. A. Atkinson, B. Koletzko, Determining life-stage groups and extrapolating nutrient intake values (NIVs). Food Nutr. Bull. 28, S61–S76 (2007). 17521120 10.1177/15648265070281S107

[16] G. Corbie-Smith, D. M. M. S. George, S. Moody-Ayers, D. F. Ransohoff, Adequacy of reporting race/ethnicity in clinical trials in areas of health disparities. J. Clin. Epidemiol. 56, 416–420 (2003). 12812814 10.1016/s0895-4356(03)00031-3

[17] Institute of Medicine (US) Standing Committee on the Scientific Evaluation of Dietary Reference Intakes and its Panel on Folate, Other B Vitamins, and Choline, *Dietary Reference Intakes for Thiamin, Riboflavin, Niacin, Vitamin B_6_, Folate, Vitamin B_12_, Pantothenic Acid, Biotin, and Choline* [National Academies Press (US), 1998]. 23193625

[18] J. K. Blusztajn, B. E. Slack, T. J. Mellott, Neuroprotective actions of dietary choline. Nutrients 9, 815 (2017). 28788094 10.3390/nu9080815 PMC5579609

[19] M. A. Caudill, B. J. Strupp, L. Muscalu, J. E. H. Nevins, R. L. Canfield, Maternal choline supplementation during the third trimester of pregnancy improves infant information processing speed: A randomized, double-blind, controlled feeding study. FASEB J. 32, 2172–2180 (2018). 29217669 10.1096/fj.201700692RR PMC6988845

[20] V. Iyengar, Nuclear and isotopic techniques for addressing nutritional problems, with special reference to current applications in developing countries. Food Nutr. Bull. 23, 3–10 (2002). 11975366 10.1177/156482650202300101

[21] D. A. Horita, S. Hwang, J. M. Stegall, W. B. Friday, D. R. Kirchner, S. H. Zeisel, Two methods for assessment of choline status in a randomized crossover study with varying dietary choline intake in people: Isotope dilution MS of plasma and in vivo single-voxel magnetic resonance spectroscopy of liver. Am. J. Clin. Nutr. 113, 1670–1678 (2021). 33668062 10.1093/ajcn/nqaa439 PMC8168360

[22] A. D. Gernand, The upper level: Examining the risk of excess micronutrient intake in pregnancy from antenatal supplements. Ann. N. Y. Acad. Sci. 1444, 22–34 (2019). 31094004 10.1111/nyas.14103 PMC6618111

[23] J. C. King, C. Garza, Harmonization of nutrient intake values. Food Nutr. Bull. 28, S3–S12 (2007). 17521115 10.1177/15648265070281S101

[24] A. L. Yaktine, J. C. King, L. H. Allen, Why the derivation of nutrient reference values should be harmonized and how it can be accomplished. Adv. Nutr. 11, 1102–1107 (2020). 32379857 10.1093/advances/nmaa048 PMC7490149

[25] J. Ioannidis, Next-generation systematic reviews: Prospective meta-analysis, individual-level data, networks and umbrella reviews. Br. J. Sports Med. 51, 1456–1458 (2017). 28223307 10.1136/bjsports-2017-097621

[26] S. He, K. C. Klatt, A. Rahnavard, M. D. Barberio, A. D. Gernand, E. R. Smith, Protocol for meta-research on the evidence informing micronutrient dietary reference intakes for pregnant and lactating women. Gates Open Res. 4, 171 (2020). 33629039 10.12688/gatesopenres.13199.1 PMC7876347

[27] L. H. Allen, A. L. Carriquiry, S. P. Murphy, Perspective: proposed harmonized nutrient reference values for populations. Adv. Nutr. 11, 469–483 (2020). 31701998 10.1093/advances/nmz096 PMC7231601

[28] National Academies of Sciences, Engineering, and Medicine; Health and Medicine Division; Food and Nutrition Board; Committee on the Development of Guiding Principles for the Inclusion of Chronic Disease Endpoints in Future Dietary Reference Intakes, *Guiding Principles for Developing Dietary Reference Intakes Based on Chronic Disease* [National Academies Press (US), 2017]. 29200241

[29] Institute of Medicine, Food and Nutrition Board, *Dietary Reference Intakes: A Risk Assessment Model for Establishing Upper Intake Levels for Nutrients* (National Academies Press, 1999). 20845565

[30] R. M. Pitkin, L. Allen, L. B. Bailey, M. Bernfield, Dietary Reference Intakes for Thiamin, riboflavin, niacin, vitamin B6, folate, vitamin B12, Pantothenic acid, biotin and choline (Washington, DC, 2000); www8.nationalacademies.org/onpinews/newsitem.aspx?RecordID=s6015. 23193625

[31] N. I. Krinsky, G. R. Beecher, R. F. Burk, A. C. Chan, j. J. W. Erdman, R. A. Jacob, I. Jialal, L. N. Kolonel, J. R. Marshall, P. R. L. Taylor Mayne, Others, Dietary reference intakes for vitamin C, vitamin E, selenium, and carotenoids, *Institute of Medicine* (2000); www8.nationalacademies.org/onpinews/newsitem.aspx?RecordID=s9810.

[32] R. M. Russell, J. L. Beard, R. J. Cousins, J. T. Dunn, G. Ferland, K. M. Hambidge, S. Lynch, J. G. Penland, A. C. Ross, B. J. Stoecker, Others, Dietary reference intakes for vitamin A, vitamin K, arsenic, boron, chromium, copper, iodine, iron, manganese, molybdenum, nickel, silicon, vanadium, and zinc, *A Report of the Panel on Micronutrients, Subcommittees on Upper Reference Levels of Nutrients and of Interpretation and Uses of Dietary Reference Intakes, and the Standing Committee on the Scientific Evaluation of Dietary Reference Intakes Food and Nutrition Board Institute of Medicine* (2001); www8.nationalacademies.org/onpinews/newsitem.aspx?RecordID=s10026.

[33] Institute of Medicine (US) Committee to Review Dietary Reference Intakes for Vitamin D and Calcium, *Dietary Reference Intakes for Calcium and Vitamin D* [National Academies Press (US), 2011]. 21796828

[34] Institute of Medicine (US) Standing Committee on the Scientific Evaluation of Dietary Reference Intakes, *Dietary Reference Intakes for Calcium, Phosphorus, Magnesium, Vitamin D, and Fluoride* [National Academies Press (US), 1997]. 23115811

[35] Institute of Medicine; Food and Nutrition Board; Panel on Dietary Reference Intakes for Electrolytes and Water, *DRI, Dietary Reference Intakes for Water, Potassium, Sodium, Chloride, and Sulfate* (National Academy Press, 2004).

[36] National Academies of Sciences, Engineering, and Medicine; Health and Medicine Division; Food and Nutrition Board; Committee to Review the Dietary Reference Intakes for Sodium and Potassium, *Dietary Reference Intakes for Sodium and Potassium* [National Academies Press (US), 2019]. 30844154

[37] Institute of Medicine, *Dietary Reference Intakes for Energy, Carbohydrate, Fiber, Fat, Fatty Acids, Cholesterol, Protein, and Amino Acids* (The National Academies Press, 2005). 10.1016/s0002-8223(02)90346-9 12449285

[38] E. R. Smith, S. He, K. Klatt, M. D. Barberio, A. Rahnavard, N. Azad, C. Brandt, B. Harker, E. Hogan, P. Kucherlapaty, D. Moradian, A. D. Gernand, H. K. Ahmadzia, Meta-research on the evidence informing micronutrient dietary reference intakes - project files 2021 (DOI 10.17605/OSF.IO/CFD67) (2021), doi:10.17605/OSF.IO/CFD67.

[39] R. C. Team, Others, R: A language and environment for statistical computing (2013); https://cran.microsoft.com/snapshot/2014-09-08/web/packages/dplR/vignettes/xdate-dplR.pdf.

