# Supplementary material for: Limited data exist to inform our basic understanding of micronutrient requirements in pregnancy
Source: Sci Adv. 2021 Oct 22;7(43):eabj8016. doi: 10.1126/sciadv.abj8016 (PMC8535830; doi:10.1126/sciadv.abj8016)
Supplement: Supplementary file 1 — Supplementary Text Figs. S1 to S4 Tables S1 to S5 [file sciadv.abj8016_sm.pdf]

Science Advances
AAAS

Supplementary Materials for

Limited data exist to inform our basic understanding of micronutrient requirements in pregnancy

Emily R. Smith*, Siran He, Kevin C. Klatt, Matthew D. Barberio, Ali Rahnavard, Negeena Azad, Carolyn Brandt, Bethany Harker, Emily Hogan, Padmini Kucherlapaty, Dina Moradian, Alison D. Gernand, Homa K. Ahmadzia

*Corresponding author. Email: emilysmith@email.gwu.edu

Published 22 October 2021, *Sci. Adv.* **7**, eabj8016 (2021)
DOI: 10.1126/sciadv.abj8016

This PDF file includes:

Supplementary Text
Figs. S1 to S4
Tables S1 to S5

Supplementary Text

Typically, there are four basic indicators comprising nutrition reference values (NRVs): 1) an estimation of the average requirement, which is the daily nutrient intake estimated to meet the needs of half the general population; 2) a population reference value, which refers to the daily dietary nutrient intake estimated to meet the nutrient needs of nearly the entire general population; 3) an estimation of adequacy, referring to the daily dietary nutrient intake *assumed* to be adequate when #1 and #2 ~~I~~ cannot be determined; 4) an estimation of an upper limit, which is the highest daily nutrient intake that is unlikely to pose a risk of harm to the general population, typically derived from a toxicological framework (*Yaktine et al. Adv Nutr 2020*).

The NRVs have been employed at the national-level as well as globally. For instance, the NRVs were used by a United Nations working group in the late 1990s to establish the dose of micronutrients included in a standard prenatal vitamin (i.e. multiple micronutrient supplements or MMS).

In addition to the Dietary Reference Intakes, the European Food Safety Authority also gives considerable time and effort to set *NRVs (European Food Safety Authority. EFSA Support Publ. 2017;14)*. Although the World Health Organization and the Food and Agriculture Organization published a set of recommended nutrient intakes, the detailed process of deriving the recommended values are not elucidated *(WHO/FAO expert consultation on human vitamin and mineral requirements. 2nd Ed. 2004; Allen et al. Adv Nutr 2020)*.

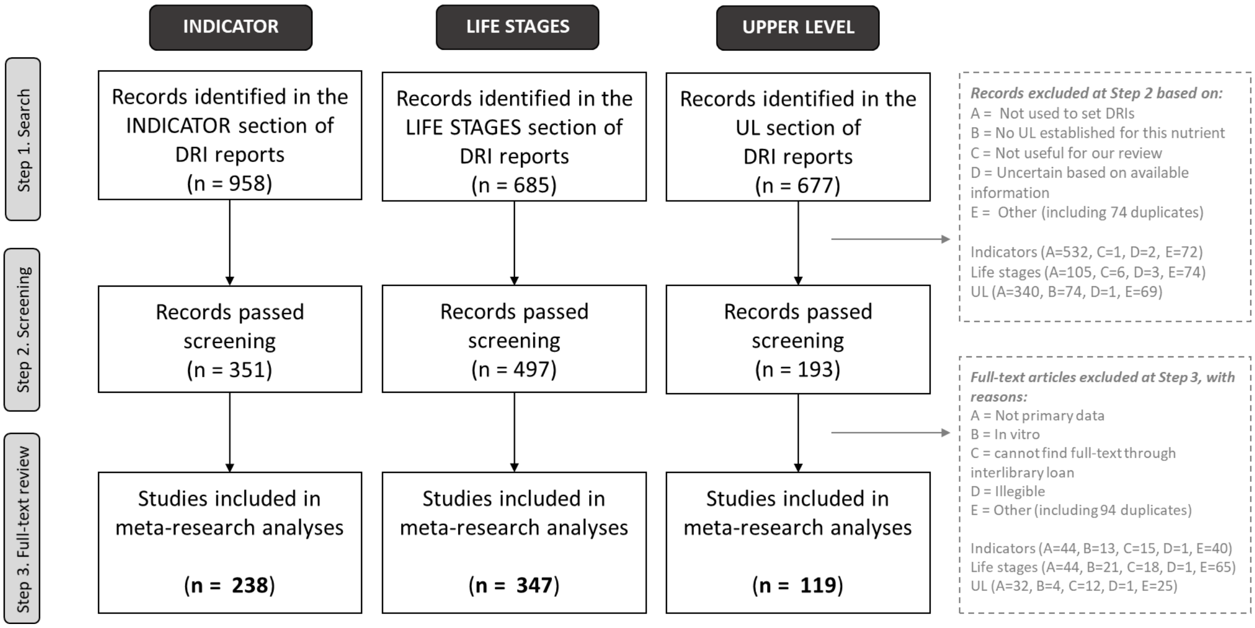

Fig. S1. Flow chart of investigated articles for all selected micronutrients.

For the “Indicator” section, we included relevant studies in the “Selection of Indicators for Estimating the Requirement for [nutrient]” section for all nutrients in the selected Dietary Reference Intake (DRI) reports, as well as the “Preeclampsia” sub-section for vitamin B6 only. For the “Life stages” section, we included “Findings by life stage and gender group” section for all nutrients in the selected DRI reports, specifically the sub-sections “Adults”, “Pregnancy”, and “Lactation”, as well as the “Approaches for deriving the estimated average requirement” section for vitamin B2 only. For the “Upper level (UL)”, we included references in the “tolerable upper intake levels” section of the reports.

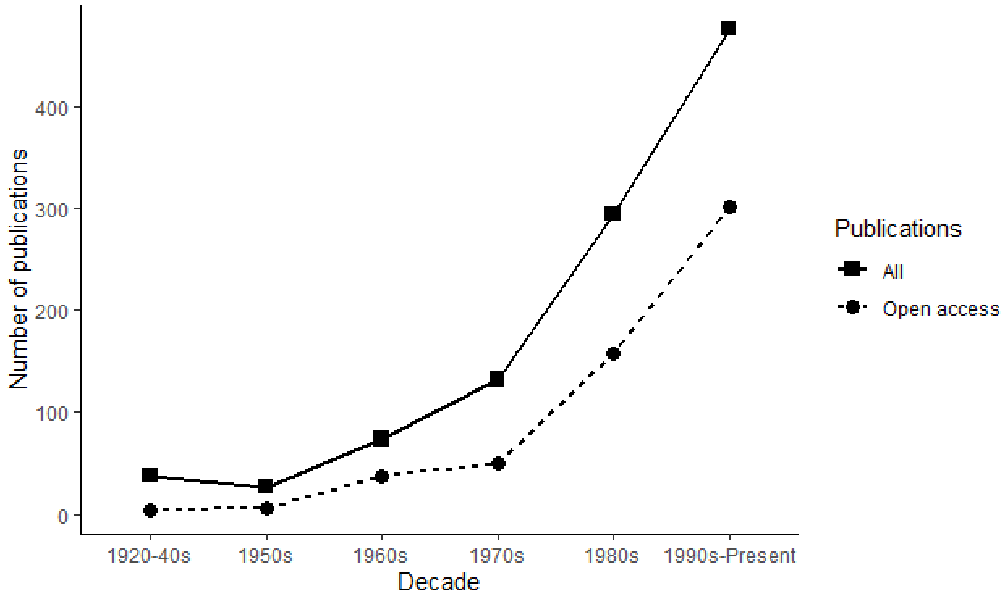

Fig. S2. Number of publications by decade for selected micronutrients, by open access status. "All" refers to all included studies in this article, and "open access" refers to articles that are distributed online, free of cost or other access barriers.

A) Inclusion of women

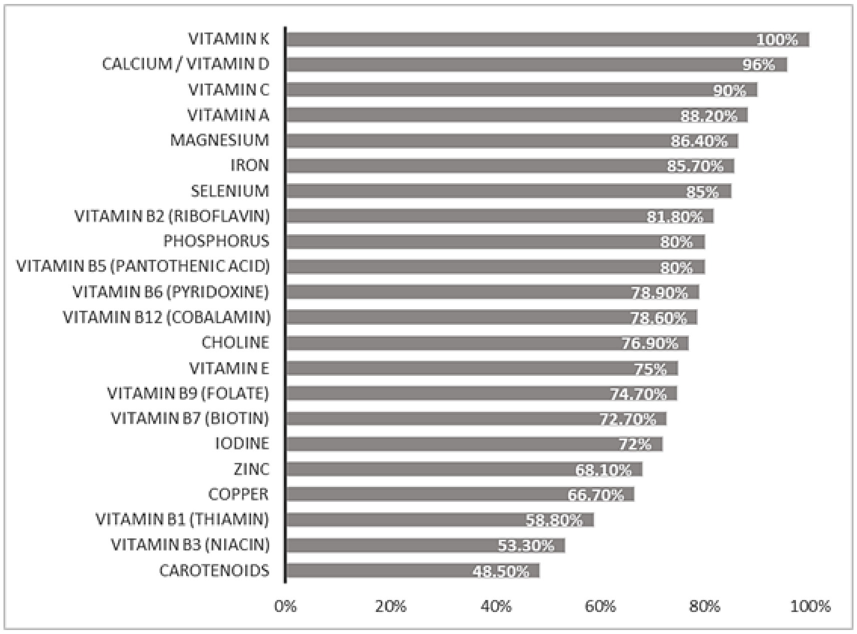

B) Inclusion of pregnant or lactating women

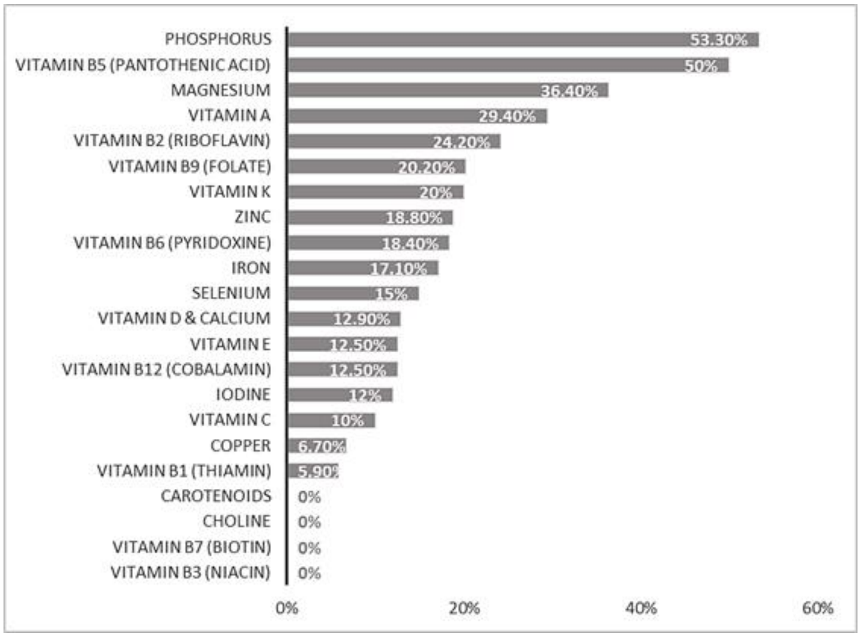

Fig. S3. Percentages of studies that included women (A) and pregnant or lactating women (B) by micronutrient in all three selected sections of the Dietary Reference Intake reports

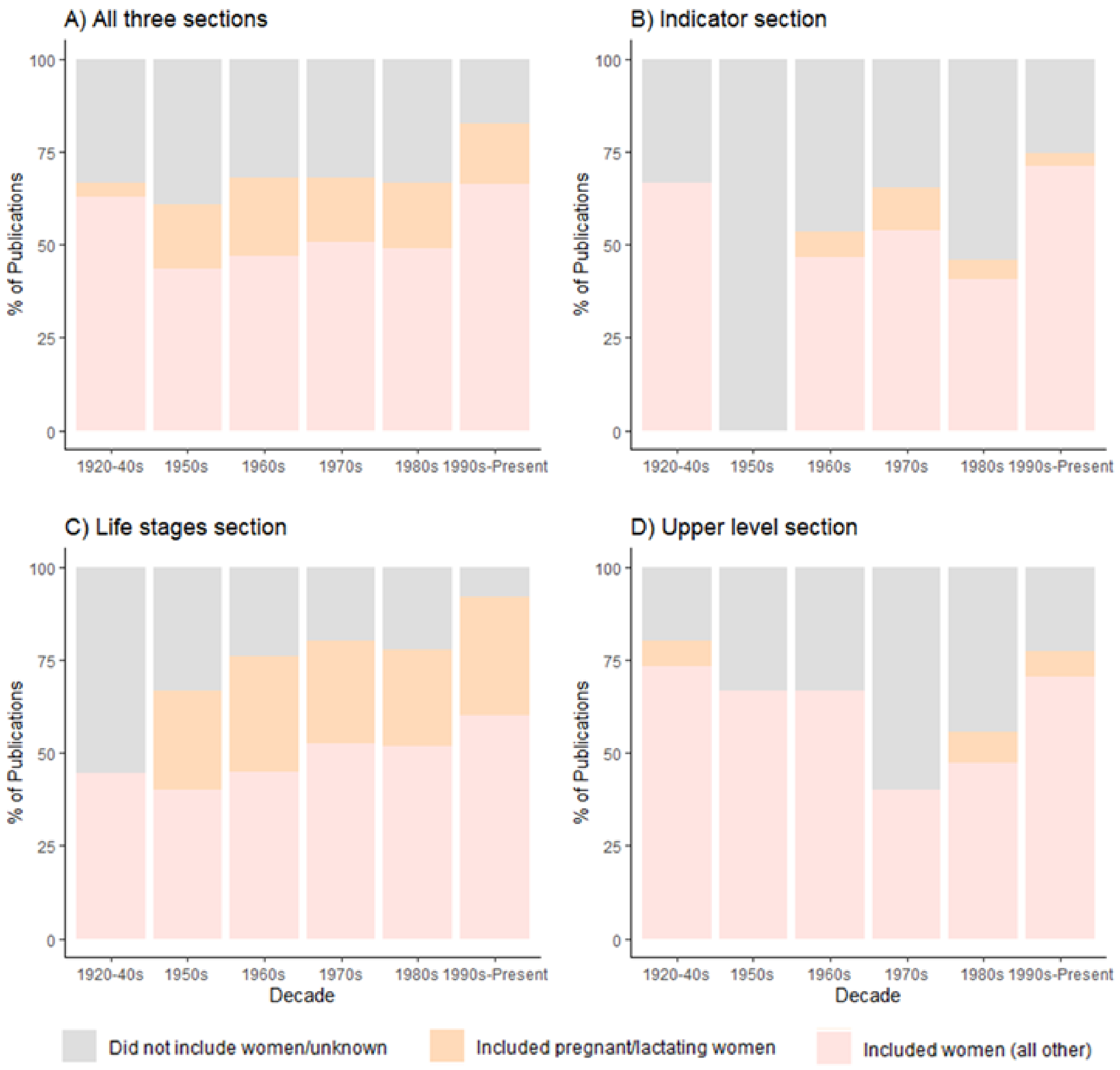

Fig. S4. Proportion of studies that included women, pregnant or lactating women by decade in the Dietary Reference Intake reports. For all three sections combined (A), proportions of studies that included women by decade (1920-40s, 50s, 60s, 70s, 80s, 90s to present) were 66.7%, 60.9%, 68.1%, 67.9%, 66.5%, and 82.5%, respectively; Proportions including pregnant or lactating women by decade were 3.7%, 17.4%, 21.3%, 17.3%, 17.5%, and 16.3%, respectively.

Table S1. Publication characteristics of the included studies [a]

Characteristics	Section in the Dietary Reference Intake report			
	**Indicator (Studies = 238)**	**Life stages (Studies = 347)**	**Upper level (Studies = 119)**	**Total (Studies = 704)**
Human study, n (%) [b]	232 (97.5%)	336 (96.8%)	102 (85.7%)	671 (95.3%)
Year of publication, range	1940 - 2008	1938 - 2010	1938 - 2009	1938 - 2010
Conducted in low- or middle-income country, n (%)	18 (7.6%)	30 (8.6%)	10 (8.4%)	58 (8.2%)
Open access, n (%)	159 (66.8%)	216 (62.2%)	48 (40.3%)	423 (60.1%)

[a] Included after step 3 full-text review

[b] When a study reported both human and non-human subjects, we reported relevant information in both sections.

Table S2. Summary of included animal studies [a]

Characteristics		Section in the Dietary Reference Intake report			
		**Indicator** (Studies = 6)	**Life stages** (Studies = 15)	**Upper level** (Studies = 18)	**Total** (Studies = 39)
Included female animals	Number of Studies	1 (16.7%)	7 (46.7%)	6 (33.3%)	14 (35.9%)
	Sample size [b]	0	258 (40.8%)	330 (37.0%)	588 (37.6%)
Included pregnant animals	Number of Studies	0	6 (40.0%)	0	6 (15.4%)
	Sample size [b]	0	56 (8.8%)	0	56 (3.6%)
Reported a shared condition or comorbidity [c]	Number of Studies	3 (50.0%)	7 (46.7%)	12 (66.7%)	22 (56.4%)

[a] Animal studies included after step 3 full-text review. Species: Rats: generic rats (not specified), albino rats, New Zealand albino rats, Sprague-Dawley rats, white rats, and Wistar rats. Cows: Holstein, Jersey and Guernsey cows. Mice: generic. Dogs included generic dogs, and purebred Beagle dogs. Bats: Egyptian fruit bats (*Rousettus aegyptiacus*). Rabbits: generic. Monkeys: Rhesus monkeys. Frogs: South African frog (*Xenopus laevis*). Mammals: “a variety of mammals”.

[b] Sample sizes of each section and overall: indicators = 39, life stages = 633, upper level = 892, total = 1,564. Not all studies reported sample size for female or pregnant animals, even when they were reported to be included

[c] Examples of shared conditions: cobalamin replete bats; cobalt-epileptic rats; all timed pregnant; all female albino rats; pathogen free mice.

Table S3. Reporting of rigorous or molecular methods in the included studies [a]

Aspects of study methods [b]		Section in the Dietary Reference Intake report			
		Indicator (Studies = 238)	Life stages (Studies = 347)	Upper level (Studies = 119)	Total (Studies = 704)
Rigor of design, n (%)	Randomized Controlled Trial	71 (29.8%)	84 (24.2%)	34 (28.6%)	189 (26.8%)
	Controlled Feeding	51 (21.5%)	68 (19.6%)	17 (14.3%)	136 (19.3%)
	Balance study	87 (36.6%)	111 (32.0%)	13 (10.9%)	211 (30.0%)
	Repeated measurements	184 (73.3%)	289 (83.3%)	94 (79.0%)	567 (80.5%)
	None above	47 (19.7%)	51 (14.7%)	22 (18.5%)	120 (17.0%)
Molecular methods, n (%) [c]	Stable isotope [d]	12 (5.0%)	18 (5.2%)	1 (0.8%)	31 (4.4%)
	Micronutrient biomarkers	198 (83.2%)	244 (70.3%)	86 (72.3%)	528 (75.0%)
	None above	39 (16.4%)	102 (29.4%)	32 (26.9%)	173 (24.6%)

[a] Included human and non-human studies after step 3 full-text review
[b] The categories and subcategories are not mutually exclusive
[c] Regarding modern or -omics methods, only one study in the “life stages” section reported using genomics methods. No other -omics methods were documented.
[d] Radioactive isotopes were used in 32 (4.5%) studies, which were not included in the stable isotope category.

Table S4. Summary of how the dietary reference intake values of selected micronutrients were established for pregnancy

Nutrient [a]	Estimated Average Requirement (EAR) or Average Intake (AI)	Tolerable Upper Intake Level (UL)
Vitamin A	EAR:<br>Lack of data in pregnancy; EAR based on vitamin A accumulation in the liver of the fetus during gestation and an assumption that the liver contains approximately half of the body's vitamin A when liver stores are low.<br>Because vitamin A in the mother's diet may be stored and mobilized later as needed and some vitamin A may be retained in the placenta, the EAR is estimated to be ~50 mcg/d in addition to the EAR for nonpregnant women.	Women of reproductive age:<br>A No Observed Adverse Effect Level (NOAEL) of approximately 4,500 μg/day of preformed vitamin A from food and supplements was based on a critical evaluation of available data.<br>Epidemiological studies evaluating the teratogenicity of vitamin A intake shortly before or during pregnancy were used to derive a UL for women of reproductive age.
Vitamin B1 (Thiamin)	EAR:<br>Despite citing several studies in the pregnancy section, these studies were deemed too equivocal to contribute to setting the EAR.<br>Therefore, for pregnancy, the requirement of thiamin is increased by ~30% based on assumptions about growth in maternal and fetal compartments.	Not determinable[b]: No reports available of adverse effects from consumption of excess thiamin by ingestion of food and supplements; Data are inadequate for a quantitative risk assessment.
Vitamin B2 (Riboflavin)	EAR:<br>For pregnancy, the requirement of riboflavin is increased by 0.3mg/day based on assumptions about growth in maternal and fetal compartments, and a small increase in energy utilization.	Not determinable[b]: No adverse effects associated with riboflavin consumption from food or supplements have been reported.
Vitamin B3 (Niacin)	EAR:<br>No direct evidence suggesting a change in the niacin requirement during pregnancy.<br>For pregnancy, the requirement of Niacin is increased by 3mg/day based on assumptions about energy utilization and growth in maternal and fetal compartments, especially in the 2nd and 3rd trimesters.	There is no evidence of adverse effects from the consumption of naturally occurring niacin in foods. The Dietary Reference Intakes report focuses on intake of niacin as a supplement, food fortification, or pharmacological agent;<br>Flushing was selected as the most appropriate endpoint on which to base a UL for niacin.
Vitamin B5 (Pantothenic acid)	AI:<br>Based on usual intake of adults and adolescents.	Not determinable[b]
Vitamin B6 (Pyridoxine)	EAR:<br>Although 0.5 mg/day of B6 may overestimate the additional need in early gestation, it was considered judicious to err on the side of ensuring sufficiency and add 0.5 mg/day to the EAR for nonpregnant women throughout pregnancy.	No adverse effects have been associated with high intake of vitamin B6 from food sources. The Dietary Reference Intakes report focuses on pyridoxine.<br>Sensory neuropathy was selected as the critical endpoint on which to base a UL for vitamin B6.

Vitamin B7 (Biotin)	AI:<br>Extrapolation from the adequate intakes of infants exclusively fed human milk	Not determinable[b]
Vitamin B9 (Folate)	EAR:<br>Based on experimental data, low dietary folate intake plus 100 µg of supplemental folate is inadequate to maintain normal folate status.<br>The EAR therefore was derived by adding this quantity (100 µg) in dietary folate equivalents (DFEs, which is 200 µg/day) to the EAR for nonpregnant women to provide an EAR of 520 µg/day of DFEs.<br>The role of folate in the prevention of neural tube defects was discussed, but was NOT used to set an EAR.	No adverse effects have been associated with the consumption of the amounts of folate normally found in fortified foods. The Dietary Reference Intakes report focuses on evidence of supplemented folate.<br>Excessive folate intake may precipitate or exacerbate neuropathy in vitamin B12-deficient individuals, which justifies the selection of this endpoint as the critical endpoint for the development of a UL for folate.
Vitamin B12 (Cobalamin)	EAR:<br>The increase for pregnancy is based on the estimation of fetal deposition of 0.1 to 0.2 µg/day throughout pregnancy, and on evidence that maternal absorption of the vitamin becomes more efficient during pregnancy; the EAR is increased by 0.2 µg/day during pregnancy.	Not determinable[b]: there is not sufficient scientific evidence to set UL for vitamin B12.
Choline	AI:<br>Based on estimations of the fetal and placental accumulation of choline.	No data to suggest additional concerns in pregnancy, so the UL is set the same as non-pregnant adults.
Vitamin C	EAR:<br>Pregnancy EAR is based on adding the EAR for near-maximal neutrophil concentration of the nonpregnant woman to the amount of vitamin C necessary to transfer adequate vitamin C to the fetus.<br>In the absence of precise data regarding transfer of maternal vitamin C to the fetus, and with the knowledge that intakes of 7 mg/day of vitamin C will prevent young infants from developing scurvy, the EAR for pregnancy was estimated to increase 10 mg/day over the vitamin C requirement for the nonpregnant woman.	No evidence of maternal toxicity of excess vitamin C intakes was found.<br>Data do warrant a separate UL for pregnant females.<br>Osmotic diarrhea and related gastrointestinal disturbances were selected as the critical endpoints on which to base a UL for vitamin C.
Vitamin D	EAR:<br>The EARs are identical across age groups, and are the same in pregnancy as well, due to the lack of an age effect on the simulated dose-response analysis.	UL for pregnant and lactating women is the same as those for their non-pregnant and non-lactating counterparts.<br>The onset of hypercalcemia and related toxicity is selected as the basis for the UL for all age groups except infants, with the caveat that it is to be subject to adjustment for uncertainty.

Vitamin E	EAR:<br>No evidence (at the time the Dietary Reference Intakes report was published) that the EAR for women during pregnancy should be increased above the level recommended for women in the nonpregnant state, the EAR for pregnancy is assumed to be the same as for non-pregnancy.	Lack of data; the UL for pregnant and lactating women is set as the same as that of non-pregnant and non-lactating women.
Vitamin K	AI:<br>The AI is based on median NHANES III intake estimates of healthy nonpregnant women.<br>Data pertaining to vitamin K status of pregnant women are limited but suggest that status is not different from that of nonpregnant women.	Not determinable[b]
Calcium	EAR:<br>The EAR for non-pregnant women and adolescents is appropriate for pregnant women and adolescents based on the randomized controlled trials (RCTs) of calcium supplementation during pregnancy.	Lacking data to suggest a basis for a different UL, the ULs for calcium for pregnancy and lactation have therefore been kept the same as those for their non-pregnant and non-lactating counterparts.
Carotenoids	N/A for EAR or AI	Not determinable[b]: A UL has not been set for β-carotene or carotenoids.<br>Instead, it is concluded that β-carotene supplements are not advisable for the general population.
Copper	EAR:<br>Lack of data; The EAR was based on estimates of the amount of copper that must be accumulated during pregnancy to account for the fetus and products of pregnancy.	A No Observed Adverse Effect Level (NOAEL) of 10 mg/day of copper was identified on the basis of the results of Pratt and coworkers in 1985.<br>Liver damage was selected as the critical endpoint on which to base a UL of copper.
Iodine	EAR:<br>The daily accumulation of iodine by the newborn was used to estimate the daily fetal iodine uptake.<br>An estimated daily thyroid iodine uptake of approximately 75 µg/day by the fetus and an EAR of 95 µg/day for nonpregnant women would yield an EAR of 170 µg/day during pregnancy.	An elevated thyroid stimulating hormone (TSH) concentration above baseline was selected as the critical adverse effect on which to base a UL for iodine.
Iron	EAR:<br>Factorial modeling is used to estimate median requirements of pregnant women with use of the equation:<br>Requirement for absorbed iron = basal losses + iron deposited in fetus and related tissues + iron utilized in expansion of hemoglobin mass.<br>The EAR and RDA are established by using estimates for the 3rd trimester to build iron stores during the 1st trimester of pregnancy.	Data are limited on GI effects in pregnant and lactating women. Due to lack of data (particularly of doses < 100mg/day), the UL for nonpregnant and nonlactating adult women was used for pregnant and lactating women.<br>Gastrointestinal side effects were selected as the critical adverse effects on which to base the UL for iron.

Magnesium	EAR:<br><br>Findings are inconsistent on magnesium supplementation and pregnancy outcomes; No data indicating that magnesium is conserved during pregnancy or intestinal absorption is increased; the natural weight gain in pregnancy may increase requirement.<br><br>Assumptions in setting this EAR in pregnancy include: appropriate gain of lean body mass; the magnesium content of lean body mass is around 470mg/1kg; and bioavailability adjustment.	The Lowest Observed Adverse Effect Level (LOAEL) identified for magnesium-induced diarrhea in adults is 360 mg (15 mmol)/day of magnesium from nonfood sources, based on experimental studies.
Phosphorus	EAR:<br><br>No evidence at this time supports an increase of the EAR and RDA during pregnancy above the level recommended during the nonpregnant state.<br><br>Slight increase based on estimates of intestinal absorption of phosphorus in pregnancy, which is considered sufficient to provide the necessary phosphorus for fetal growth.	As the primary initial manifestation of excessive magnesium intake, diarrhea was selected as the critical endpoint.<br><br>No evidence suggests increased susceptibility to adverse effects of supplemental magnesium during pregnancy and lactation, hence the UL is the same as non-pregnant and non-lactating women.
Selenium	EAR:<br><br>Lack of data; the pregnancy EAR is set to allow accumulation of enough selenium by the fetus to saturate its selenoproteins.<br><br>Based on a fetal deposition of 4 μg/day throughout pregnancy, the EAR is increased by this amount during pregnancy.	No reports of teratogenicity or selenosis in infants born to mothers with high but not toxic intakes of selenium. Therefore, ULs for pregnant and lactating women are the same as for nonpregnant and nonlactating women.
Zinc	EAR:<br><br>Women's EAR is based on men's values (multiple by 0.86 to adjust for the different average surface area of women, and accordingly the average total zinc endogenous losses are 0.46 mg/ day for women)<br><br>Pregnant women's EAR is based on the additional requirement during the fourth quarter (2.7 mg/day) of pregnancy plus the EAR for nonpregnant adolescent girls and women.	The UL of 40 mg/day is based on reduction in erythrocyte copper-zinc superoxide dismutase activity.<br><br>The adverse effect of excess zinc on copper metabolism was chosen as the critical effect on which to base a UL for total daily intake of zinc from food, water, and supplements in humans.

[a] The order of the micronutrients are alphabetical, for vitamins first, followed by minerals and trace elements.

[b] Not determinable: due to lack of data of adverse effects in this life stage group, and concern with regard to lack of ability to handle excess amounts.

Table S5. Summary of funding agencies and conflict of interest in included studies

Funding source	Government only	Private industry only	Foundation / philanthropic organizations only	Other sources / Mixed sources	Did not report funding
All	326	31	44	97	206
Included women	240 (73.6%)	28 (90.3%)	31 (70.5%)	75 (77.3%)	146 (70.9%)
Included pregnant or lactating women	54 (16.6%)	6 (19.4%)	3 (6.8%)	11 (11.3%)	43 (20.9%)
Examples of specific funding agencies [a]	• Medical Research Council (e.g., South Africa, India, Sweden)<br>• US National Institutes of Health<br>• The World Health Organization	• F. Hoffmann La-Roche & Co. Ltd.<br>• Johnson & Johnson Corp.<br>• Kellog Inc.	• Foundation for Pediatric Research, Finland<br>• Rockefeller Foundation<br>• The Wellcome Trust	• Personal donations from the macrobiotic community<br>• Combinations of the three categories to the left	N/A

[a] For a full list of funding agencies in the included studies, please refer to the study data at the repository https://osf.io/cfd67/ (variable names: "Funding_type" and "Funding_text").
